# Laryngeal Lymphoma: The High and Low Grades of Rare Lymphoma Involvement Sites

**DOI:** 10.1155/2014/284643

**Published:** 2014-07-22

**Authors:** Charles Paul Azzopardi, James Degaetano, Alexandra Betts, Eric Farrugia, Claude Magri, Nicholas Refalo, Alexander Gatt, David J. Camilleri

**Affiliations:** ^1^Department of Haemato-Oncology, Mater Dei Hospital, Tal-Qroqq, Msida MSD 2090, Malta; ^2^Pathology Department, Mater Dei Hospital, Tal-Qroqq, Msida MSD 2090, Malta; ^3^Department of Surgery, Mater Dei Hospital, Tal-Qroqq, Msida MSD 2090, Malta

## Abstract

The larynx is an extremely rare site of involvement by lymphomatous disease. We present two cases of isolated laryngeal high-grade and another low-grade lymphoma, together with a literature review of laryngeal lymphoma management.

## 1. Case 1

**Figure 1:**
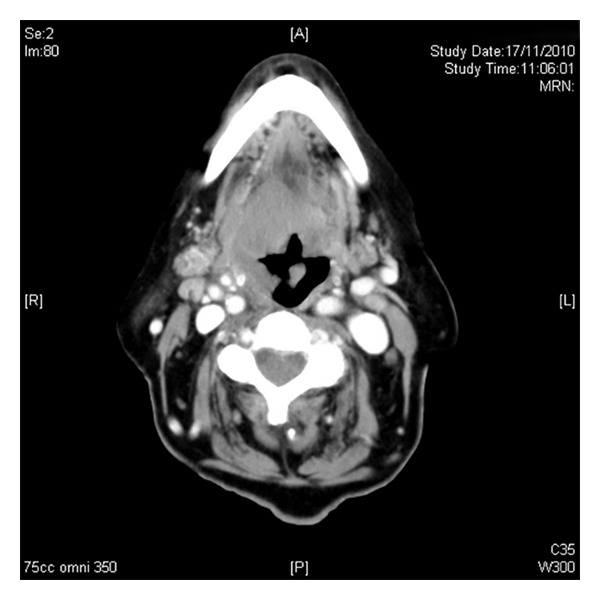
Computed tomography at presentation.

An 82-year-old Caucasian lady known to suffer from ischaemic heart disease, hypertension, and mild renal impairment was admitted to hospital in November 2010 complaining of a 3-month history of increasing facial pain and difficulty breathing. She had previously undergone surgery for a complicated diverticular abscess two years prior to this presentation and this had resulted in a defunctioning colostomy. Physical examination showed no lymphadenopathy or organomegaly. Indirect laryngoscopy showed a mass at the base of the tongue, involving the epiglottis and causing airway obstruction. The patient underwent an emergency tracheostomy to secure the airway. Investigations showed a white blood count of 11.3 × 10^9^/L, haemoglobin of 104 g/L, platelets of 296 × 10^9^/L, and a normal serum lactate dehydrogenase level of 209 U/L. Staging with computed tomography only confirmed disease in the laryngeal area (Figure 1) and did not show any thoracic, abdominal, or pelvic lymphadenopathy. Bone marrow aspiration and biopsy showed no evidence of bone marrow involvement.

**Figure 2:**
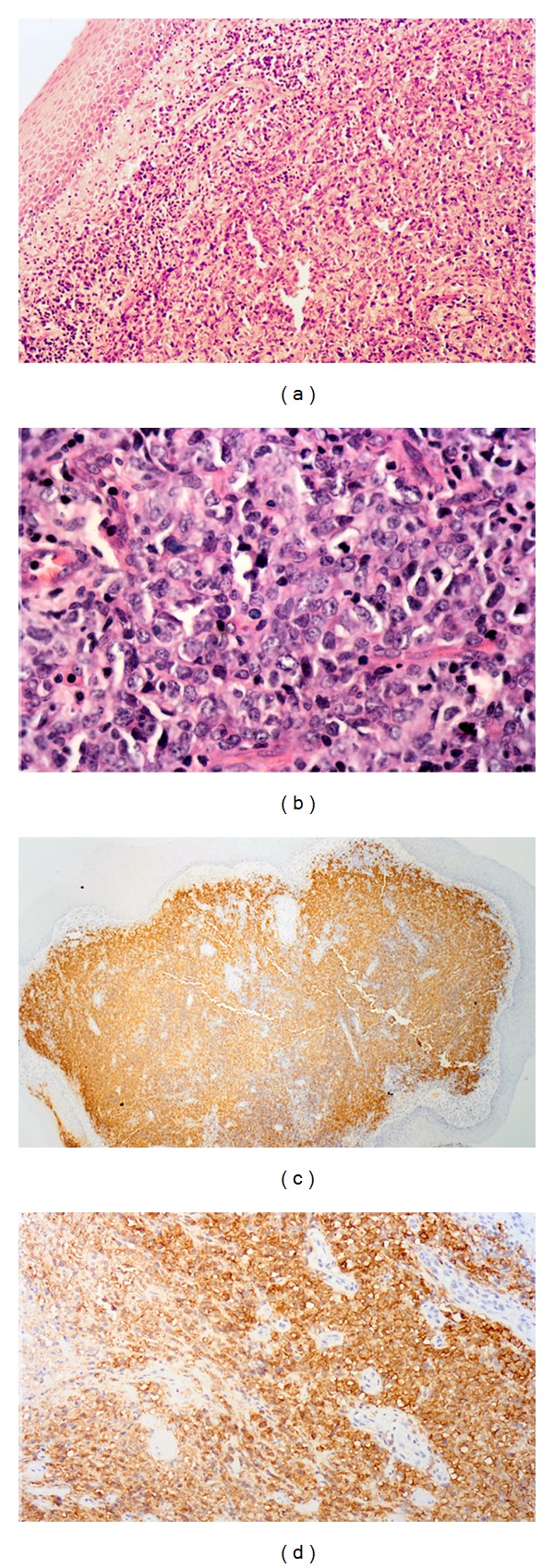
Infiltrate of vocal cord by large lymphoid cells on H&E ((a) and (b)), positive for CD20 ((c) and (d)) on immunohistochemistry.

Biopsy of the laryngeal mass showed that the mucosa and submucosa were infiltrated by large atypical lymphoid cells with irregular nuclear outlines and multiple prominent nucleoli. The cells were positive for CD45 and CD20 and negative for CD3 and bcl2 on immunohistochemistry (Figure 2). These findings were consistent with a diagnosis of diffuse large B-cell (high-grade) non-Hodgkin lymphoma of the larynx, stage I_E_, with an R-IPI score of 1 (age > 60, ECOG 0–2, normal LDH of 209 U/L, 0-1 extranodal sites, and stage I/II disease).

The patient was administered three cycles of R-CEOP (rituximab, cyclophosphamide, etoposide, vincristine, and prednisone) followed by involved field radiotherapy. Anthracyclines were avoided in view of a decreased left ventricular ejection fraction of 40% on echocardiography performed at diagnosis.

The first cycle of chemotherapy was complicated by an acute coronary syndrome, from which the patient recovered with supportive care. The tracheostomy was removed following the second cycle of cytotoxics and the patient remains alive and well 5 years later.

## 2. Case 2

A 65-year-old Caucasian gentleman, with a history of ischaemic heart disease, presented to ENT with a two-month history of hoarseness. Physical examination was unremarkable, with no lymphadenopathy or organomegaly. Investigations showed a white blood count of 5.7 × 10^9^/L, haemoglobin of 148 g/L, platelets of 213 × 10^9^/L, and a normal serum lactate dehydrogenase level of 192 U/L.

**Figure 3:**
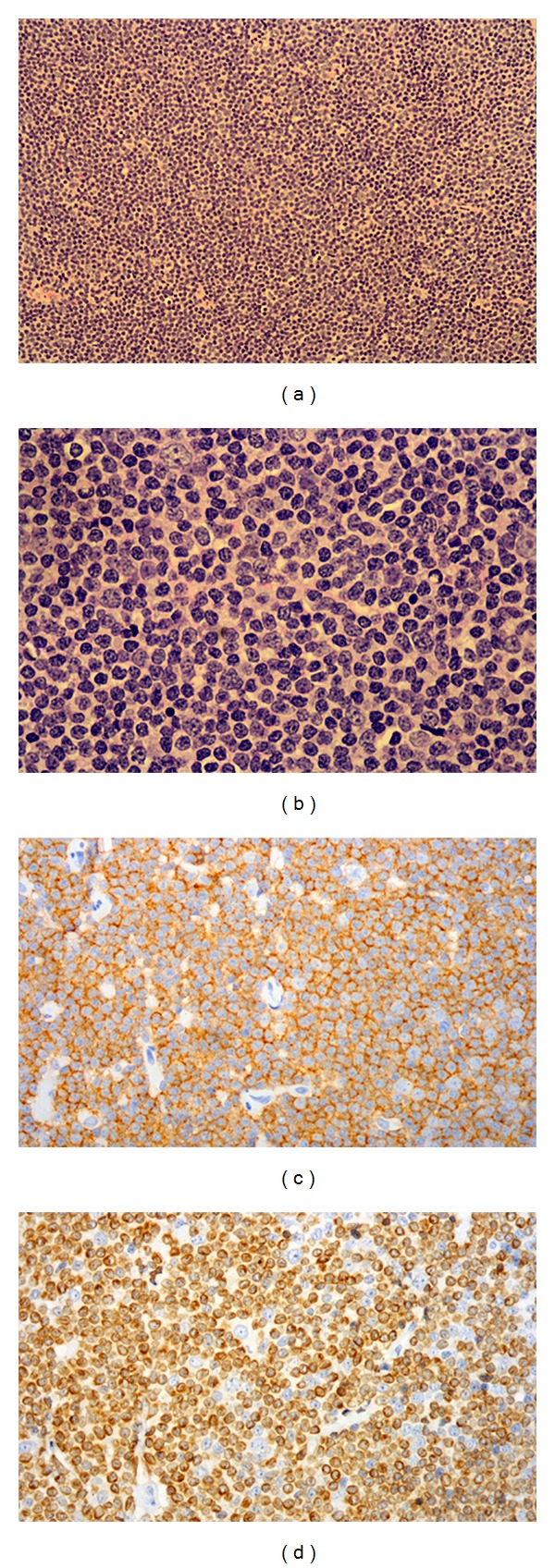
Infiltrate of vocal cord by small sized lymphoid cells on H&E ((a) and (b)), positive for CD20 (c) and bcl-2 (d) on immunohistochemistry.

Indirect laryngoscopy showed vocal polyps, which were excised. Histology showed a monotonous population of lymphoid cells within the lamina propria of mucosal polyps, with slightly irregular nuclear contours and inconspicuous to clear cytoplasm. Immunohistochemistry was positive for CD45, CD20, and bcl2 and negative for CD5, CD23, bcl6, CD138, CD43, CD10, and cyclin D1 (Figure 3), consistent with a diagnosis of extranodal marginal zone (low-grade) non-Hodgkin lymphoma.

Staging with computed tomography and bone marrow aspiration and biopsy showed no evidence of disease involvement outside of the larynx.

The patient was managed with involved field radiotherapy to the larynx with clinical resolution of symptoms and no radiological evidence of residual disease. He remains alive and well 5 years later.

## 3. Discussion

Extranodal lymphomas confined to the larynx are rare, accounting for <1% of all laryngeal neoplasms, with only about 100 cases having been described in the literature to date [1]. A ten-year review involving 2631 laryngeal biopsies in a large Spanish hospital revealed only one case of diffuse large B cell lymphoma [2]. This rare occurrence is due to the relatively low lymphoid content in the larynx when compared to other areas in the respiratory tract. The mean age at diagnosis is 70 years, with a range from 4 to 81 years. The male : female ratio has been reported to be variable in different series [3–5].

Laryngeal lymphoma presents clinically in a similar fashion to squamous cell carcinoma, with symptoms such as hoarseness, dyspnoea, a foreign body sensation in the throat, or stridor. Uncommonly, it may present catastrophically with acute airway obstruction requiring immediate surgical intervention, as in Case 1 presented above. Systemic symptomatology is unusual, since laryngeal lymphomas tend to remain localized for prolonged periods, though more aggressive forms tend to spread earlier [6]. Interestingly, these tumors usually spread to other mucosal sites such as bowel, lung, and orbit rather than nodal sites [7, 8].

The commonest anatomical site involved is the supraglottic region (47%), with glottic involvement accounting for 25% of cases. The transglottic and subglottic regions are much less commonly affected [6].

Macroscopically, these tumors present as smooth or polypoid masses as depicted in our two cases, respectively, rather than ulcerated masses [6]. In fact, both the macroscopic and radiological appearances of a large laryngeal tumour with a supraglottic submucosal component should alert the reporting investigators to the possibility of laryngeal lymphoma [3–5], though the definite diagnosis always rests on histology. Histologically, primary laryngeal lymphoma is more commonly of B-cell origin, though some T-cell and NK-cell lymphomas may occur. The latter are more difficult to diagnose and usually require deep and sometimes repeated biopsy. This latter subgroup of laryngeal lymphoma is commoner in HIV patients. The B-cell to T/NK-cell Lymphoma ratio is 6 : 1 [1, 5, 7].

There is some emerging evidence in the literature of the association of* H. pylori *and other urease-splitting organisms, which may colonize the larynx, and the development of Primary Marginal Zone Laryngeal Lymphoma. When investigating Primary Marginal Zone Laryngeal Lymphoma one should also exclude autoimmune conditions such as Sjögren's syndrome [7, 8].

It is imperative to stage the lymphomatous process correctly, since treatment varies depending on both the grade and the stage of the disease. PET-CT, in particular, is finding an important place for radiological staging in laryngeal lymphoma, both low-grade and high-grade [9, 10]. In our cases we did not perform PET-CT since these cases predated the advent of this radiological modality in our country.

A review of the literature on isolated laryngeal lymphomas published over the last 2 years (from 1994–2014) mainly revealed case reports and reviews. No specific studies have been carried out specifically looking at the management of this rare type of lymphoma. Most reports described cases of MALT lymphomas as in our Case 2 and others related to T/NK lymphomas [11]. Therapy was nonhomogenous due to the different types of lymphomas as well as small numbers. However, the main modalities of treatment were IFRT (30–50 G) alone or in combination with chemotherapy. We opted for 3 cycles of chemoimmunotherapy for Case 1 with DLBCL and IFRT in Case 2 with MALT according to international guidelines on limited stage disease [12]. Surgical intervention is only usually required in cases presenting with acute airway obstruction [6]. This disparity in treatment modalities, independent of lymphoma histology, is depicted in a table of case series reports found in the literature, spanning the period of 1986 to 2013, presented by Bayoumi et al. [11]. A combined chemotherapy-radiotherapy, seems to be, however, the emerging preferred modality of treatment, especially for high-grade lymphomas [6, 11].

From this review, the most important points to emerge are as follows.

1. One should have a high index of suspicion for lymphoma of the larynx, since the management is very different from that of other more commonly occurring tumors at this site.
2. Due to the small number of cases reported, there is no definite consensus regarding best management of laryngeal lymphoma; it would be advisable that for the time being one follows international lymphoma guidelines on limited stage disease for the management of laryngeal lymphoma [12].
3. The discussions ensuing from case reports regarding lymphoma affecting unusual sites highlight the lack of evidence regarding the actual biology of these tumors and best treatment options for patients. This may spur the formation of regional/international databases for the description of lymphomas affecting specific unusual sites. With this approach these cases may be studied in greater depth and may prompt the development of comparative treatment trials, providing patients with more evidence-based therapeutic modalities.
